# Identification of DNA hypermethylation of *SOX9* in association with bladder cancer progression using CpG microarrays

**DOI:** 10.1038/sj.bjc.6604143

**Published:** 2007-12-18

**Authors:** A Aleman, L Adrien, L Lopez-Serra, C Cordon-Cardo, M Esteller, T J Belbin, M Sanchez-Carbayo

**Affiliations:** 1 Tumor Markers Group, Molecular Pathology Program, Spanish National Cancer Center, Madrid, Spain; 2 Department of Pathology, Albert Einstein College of Medicine, Bronx, NY, USA; 3 Epigenetics Group, Molecular Pathology Program, Spanish National Cancer Center, Madrid, Spain; 4 Division of Molecular Pathology, Memorial Sloan-Kettering Cancer Center (MSKCC), New York, NY, USA

**Keywords:** bladder cancer, CpG arrays, methylation

## Abstract

CpG island arrays represent a high-throughput epigenomic discovery platform to identify global disease-specific promoter hypermethylation candidates along bladder cancer progression. DNA obtained from 10 pairs of invasive bladder tumours were profiled *vs* their respective normal urothelium using differential methylation hybridisation on custom-made CpG arrays (*n*=12 288 clones). Promoter hypermethylation of 84 clones was simultaneously shown in at least 70% of the tumours. *SOX9* was selected for further validation by bisulphite genomic sequencing and methylation-specific polymerase chain reaction in bladder cancer cells (*n*=11) and primary bladder tumours (*n*=101). Hypermethylation was observed in bladder cancer cells and associated with lack of gene expression, being restored *in vitro* by a demethylating agent. In primary bladder tumours, *SOX9* hypermethylation was present in 56.4% of the cases. Moreover, *SOX9* hypermethylation was significantly associated with tumour grade and overall survival. Thus, this high-throughput epigenomic strategy has served to identify novel hypermethylated candidates in bladder cancer. *In vitro* analyses supported the role of methylation in silencing *SOX9* gene. The association of *SOX9* hypermethylation with tumour progression and clinical outcome suggests its relevant clinical implications at stratifying patients affected with bladder cancer.

Epigenetic changes and genetic mutations contribute significantly to the onset of human malignancies (Jones and Baylin, 2002; Feinberg *et al*, 2006). The most common epigenetic event in the human genome is the addition of methyl groups to the carbon-5 position of cytosine nucleotides (Costello *et al*, 2000; Esteller *et al*, 2001; Esteller, 2002; Jones and Baylin, 2002; Feinberg *et al*, 2006). CpG islands are present in one-half of the human genes, and typically overlap with promoters and first exons of genes (Costello *et al*, 2000; Esteller *et al*, 2001; Esteller, 2002; Jones and Baylin, 2002; Feinberg *et al*, 2006). Hypermethylation of CpG islands is frequently associated with inappropriate transcriptional silencing of critical genes including tumour suppressors (Costello *et al*, 2000; Esteller *et al*, 2001; Esteller, 2002; Jones and Baylin, 2002; Feinberg *et al*, 2006). Transcriptional inactivation by CpG island promoter hypermethylation is a well-established mechanism for gene silencing in bladder cancer (Cordon-Cardo *et al*, 2000; Muto *et al*, 2000; Lee *et al*, 2001; Markl *et al*, 2001; Stoehr *et al*, 2004; Catto *et al*, 2005; Chapman *et al*, 2005; Kim *et al*, 2005; Marsit *et al*, 2005; Wolff *et al*, 2005; Urakami *et al*, 2006). Genes reported to be epigenetically inactivated by hypermethylation in bladder cancer include *p16*^*INK4a*^, *CDKN2A*, *RUNX3 or RASSF1*, among others (Cordon-Cardo *et al*, 2000; Muto *et al*, 2000; Lee *et al*, 2001; Markl *et al*, 2001; Stoehr *et al*, 2004; Catto *et al*, 2005; Chapman *et al*, 2005; Kim *et al*, 2005; Marsit *et al*, 2005; Wolff *et al*, 2005; Urakami *et al*, 2006).

CpG island arrays represent a high-throughput technology for the discovery of CpG island loci frequently hypermethylated during disease progression (Yan *et al*, 2000; Ching *et al*, 2005). Gene profiling analyses of cancer cells after exposure to demethylating agents may also uncover critical genes susceptible to be epigenetically silenced (Liang *et al*, 2002). Using a high-throughput epigenomic approach with CpG arrays, we identify novel candidates such as *SOX9*, presenting CpG island promoter hypermethylation in bladder cancer. To the best of our knowledge, *SOX9* had not been reported to be epigenetically modified, nor differentially expressed in bladder malignancies. In bladder cancer cell lines, the low expression of *SOX9* was restored by a demethylating agent in methylated cancer cells. Furthermore, SOX9 promoter hypermethylation was frequently found in bladder tumours. Moreover, *SOX9* methylation was associated with tumour grade and overall survival of bladder cancer patients. These are relevant findings not only as they relate to cancer progression but also for their utility in the clinical management of patients affected by uroepithelial neoplasias.

## METHODS

### Tumour samples, cell lines, genomic DNA and RNA extraction

Following IRB-approved protocols at MSKCC, primary bladder tumours and paired normal urothelium were frozen in liquid nitrogen immediately after resection and stored at −80°C until processing. Bladder tissues embedded in OCT were macrodissected to ensure a minimum of 75% of tumour or normal urothelium counterpart cell subpopulations (Sanchez-Carbayo *et al*, 2006). Genomic DNA was extracted using a non-organic method (Oncor, Gaithersburg, MD, USA). Total RNA was isolated in two steps using TRIzol (Life Technologies, Carlsbad, CA, USA), followed by RNeasy purification (Qiagen, Valencia, CA, USA). DNA and RNA quality was evaluated based on 260/280 ratios of absorbances and the integrity was also checked by gel electrophoresis analysis using the Agilent 2100 Bioanalyzer (Agilent Technologies, Palo Alto, CA, USA). For CpG methylation arrays, invasive bladder tumours (*n*=10) were compared to their paired normal urothelium. An independent series of 101 bladder tumours (non-muscle invasive (*n*=56): pTa (*n*=24), pT1 (*n*=32); and muscle invasive (*n*=45): pT2 (*n*=16), pT3 (*n*=25), pT4 (*n*=4)) was utilised to validate the relevance of methylation candidates in bladder cancer progression (Sanchez-Carbayo *et al*, 2006). Human bladder cancer cell lines (*n*=11) were obtained from the American Type Culture Collection (Rockland, MD, USA) (Sanchez-Carbayo *et al*, 2002) and from the European Collection of Cell Cultures (Salisbury, United Kingdom). This set of cell lines included RT4, 5637, J82, T24, UM-UC-3, HT1376, HT-1197, TCCSUP, ScaBER, SW780 and RT112.

### CpG island arrays

Hypermethylation patterns of genomic DNA obtained from 10 pairs of bladder tumours were profiled *vs* their respective normal urothelium using differential methylation hybridisation as previously reported (Yan *et al*, 2002; Adrien *et al*, 2006). A panel of 12 288 CpG island clones was created from the CGI genomic library from Cross *et al*, 1994, as part of the Human Genome Mapping Project Resource Center (Heisler *et al*, 2005). Inserts from this CGI library were polymerase chain reaction (PCR)-amplified, purified by ethanol precipitation and then arrayed onto glass microscope slides by a specialised robot designed and created at Albert Einstein College of Medicine (Cheung *et al*, 1999; Adrien *et al*, 2006). The Differential Methylation Hybridisation (DMH) technique was carried out as previously described (Yan *et al*, 2002). Briefly, genomic DNA was initially digested with the four-base (TT^AA) restriction enzyme *Mse*I at 37°C. This digestion restricted most genomic DNA into fragments less than 200 bp in length, and left the GC-rich CpG islands relatively intact. Experimental DNA fragments were ligated to linker primers using the FastLink DNA ligation kit (Epicentre, Madison, WI, USA). Samples were then digested with two methylation-sensitive restriction enzymes (*Bst*UI and *Hpa*II) to increase coverage of the genome and to ensure complete digestion. The resulting DNA digests were purified and subsequently amplified by PCR. Using this approach, genomic DNA fragments containing unmethylated CpG sites in one sample (e.g. the reference normal urothelium specimen) were degraded by restriction digests and not be amplified. However, corresponding DNA fragments in the other sample (the bladder tumour specimen) that contain methylated restriction sites were protected from enzyme digestion and subsequently amplified by PCR. Differentially methylated sequences were identified by comparing hybridisation signals between fluorescently labelled tumour (Cy5) and reference (Cy3) amplicons when hybridised to the CpG microarray. Fluorescent labelling of DNA amplicons was carried out using the Klenow fragment of DNA polymerase (Fisher, Pittsburg, PA, USA) with random hexamers (Invitrogen, Carlsbad, CA, USA). Hybridisation to CpG arrays was carried out overnight at 50°C in a buffer containing 30% formamide, 3 × SSC, 0.75% SDS and 100 ng of human Cot-1 DNA. Following hybridisation, slides were briefly washed with a solution of 1 × SSC, 0.1% SDS, then washed for 20 min at room temperature in 0.2 × SSC, 0.1% SDS and 20 min at room temperature in 0.1 × SSC (without SDS). Slides were immediately dried as before and scanned using the GenePix 4000A microarray scanner (Molecular Devices, Sunnyvale, CA, USA).

### Methylation analyses of the promoter of *SOX9*

The CpG island methylation status of this gene was analysed by two PCR analysis strategies of bisulphite-modified genomic DNA, which induces chemical conversion of unmethylated, but not methylated, cytosine to uracil. First, methylation status was analysed by genomic sequencing of both strands of their promoters after bisulphite treatment of genomic DNA of at least eight clones per each of the bladder cancer cell lines, as previously reported (Paz *et al*, 2003). Confirmation on at least two independent clones was required to assign methylation sites. A second strategy used methylation-specific PCR (MS-PCR) with primers specific for either the methylated or the modified unmethylated DNA. Primer sequences for bisulphite sequencing and unmethylated and methylated reactions were designed encompassing their transcription start sites distant at 723 bp before the ATG starting codon. Table 1 summarises primers, annealing temperatures and conditions for both approaches. DNA from normal lymphocytes treated *in vitro* (IVD) with *Sss*I methyltransferase was used as a positive control for methylated alleles. DNA from normal lymphocytes was used as a positive control for unmethylated alleles. PCR products were loaded onto nondenaturing 2% agarose gels, stained with ethidium bromide and visualised under an ultraviolet transilluminator.

### Analysis of *SOX9* expression in bladder cancer cell lines

Cell lines were treated with 1 and 5 *μ*M 5-AZA-2′-deoxycytidine (AZA; Sigma, St Louis, MO, USA) for 72 h to achieve demethylation (Herman and Baylin, 2003; Paz *et al*, 2003). RNA was isolated using RNeasy kit from Qiagen (Life Technologies, Gaithersburg, MD, USA). RNA (1 *μ*g) was reverse-transcribed using AMV Reverse Transcriptase (Promega, Madison, WI, USA) and amplified using specific primers and conditions for *SOX9* (Table 1). PCR was performed using a final volume of 15 *μ*l containing 1 × PCR Ecostart buffer (Ecogen, Barcelona, Spain), 1.5 mM of MgCl_2_, 0.2 mM of dNTP, 0.25 *μ*M of each primer and 1.5 U of Ecostart *Taq* polymerase (Ecogen). For PCR amplification, 0.4 *μ*g of cDNA was utilised. Reverse transcription polymerase chain reaction (RT—PCR) primers were designed between different exons and encompassing large introns to avoid any amplification of genomic DNA. Glyceraldehyde-3-phosphate dehydrogenase was used as an internal control to ensure cDNA quality and loading accuracy. The amplification products were resolved by 2% agarose gel electrophoresis and visualised by ethidium bromide staining. Cell lysates for protein analysis were analysed by western blotting using an anti-SOX-9 antibody (goat polyclonal; BD, 1/250 dilution). Equal loading was tested by re-probing with an antibody against human *α*-tubulin (mouse monoclonal; Sigma, Saint Louis, MI, 1/4000 dilution). Gels were cast using the Miniprotean 3 system (Biorad, Hercules, CA, USA) and developed using ECL immunodetection reagents (Amersham Pharmacia Biotech, Piscataway, NJ, USA).

### Immunofluorescence

Cells were grown on coverslips in P6 dishes, fixed in 4% formaldehyde and fluorescently stained (Herman and Baylin, 2003; Paz *et al*, 2003). To monitor AZA exposure, cells were stained for *SOX9* at 1/400 dilution for 45 min, using the antibody mentioned above. The secondary antibody was used at 1/250 dilution. Confocal optical sections were obtained using a Leica TCS SP microscope (Leica Microsystems, Wetzlar, Germany) equipped with krypton and argon lasers. Images were acquired and processed using the Leica LCS Lite software.

### Statistical analysis

Associations among methylation patterns of *SOX9* with clinicopathological variables such as tumour stage and tumour grade were evaluated using nonparametric Wilcoxon–Mann–Whitney and Kruskall–Wallis tests (Dawson-Saunders and Trapp, 1994). Associations of methylation patterns with survival were evaluated in those cases for which follow-up information were available, using the log-rank test (Dawson-Saunders and Trapp, 1994). Overall survival time was defined as the years elapsed between surgery and death from disease (or the last follow-up date). Patients who were alive at the last follow-up or lost were censored. Survival curves were plotted using Kaplan–Meier methodology. Statistical analyses were performed using the SPSS statistical package (version 8.0) (SPSS Inc., Chicago, IL, USA).

## RESULTS

### Identification of methylated candidates in bladder cancer progression using CpG island arrays

**Figure 1:**
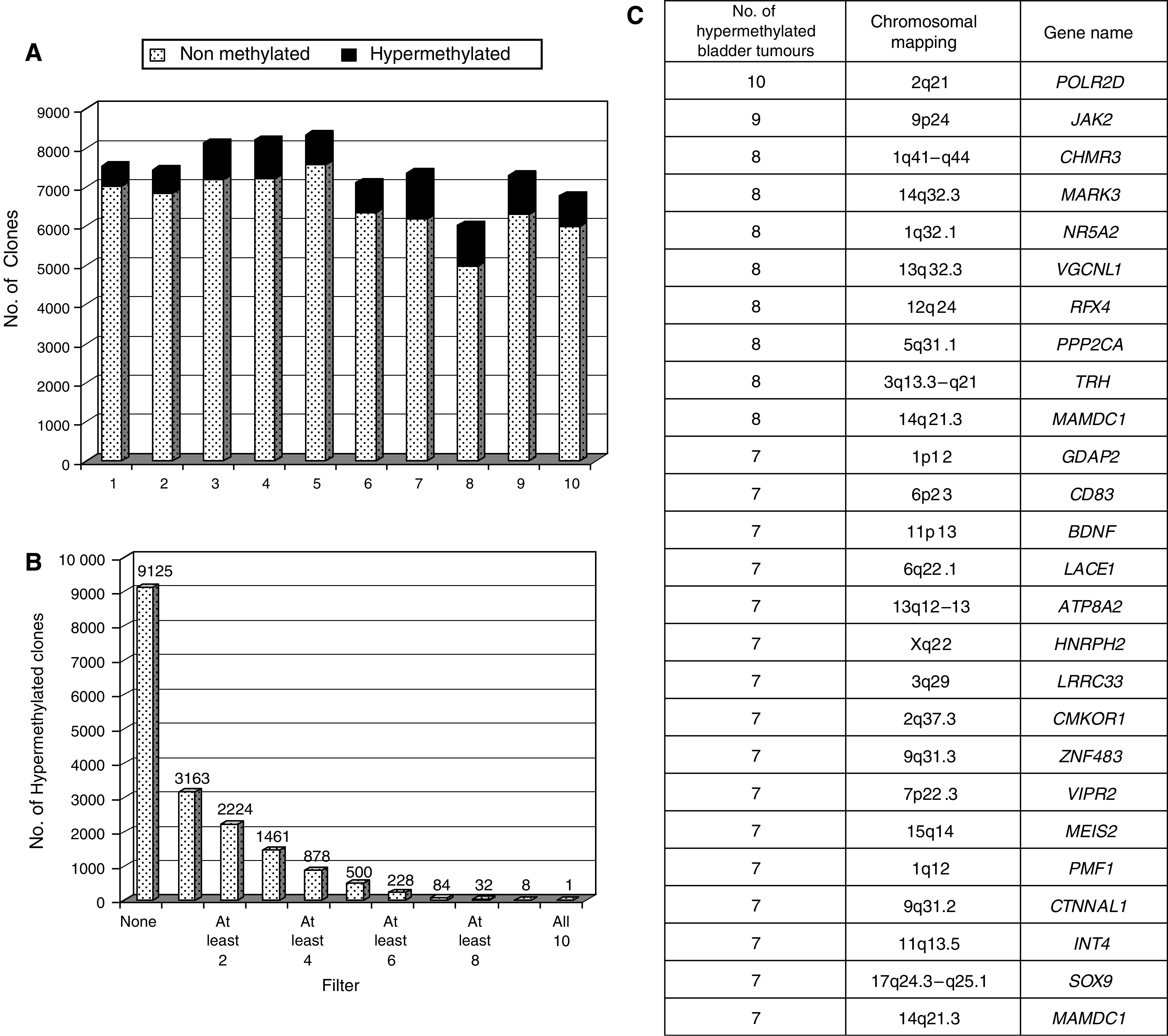
CpG arrays. (**A**) Quality control: the relative percentage of methylated and unmethylated genes was similar among the pairs of bladder tumours and normal urothelium samples under study. (**B**) CpG island arrays identify hypermethylated candidates in patients with bladder cancer. (**C**) Summary of known genes among the 84 clones simultaneously hypermethylated in 7 out of 10 samples under analysis. The number of cases found differentially expressed with a Cy5/Cy3 ratio higher than 2 for these genes are also indicated.

For 10 bladder cancer patients, differential methylation hybridisation was utilised on a CpG island microarray to identify aberrantly methylated genes in advanced bladder tumour specimens (Cy5) as compared to their corresponding normal urothelium counterpart (Cy3). Among the 12 288 CpG islands analysed, half of them provided informative results. The similar rates of hypermethylated and informative clones among the 10 pairs of bladder tumours *vs* their normal urothelium supported a similar and optimal performance of the DNA handling, labelling and hybridisation of the CpG island arrays (Figure 1A). Between 511 and 1186 clones were identified to be individually hypermethylated in each tumour sample relative to the corresponding normal urothelium tissue, based on a Cy5/Cy3 ratio of 2.0 or greater (Figure 1A). Among the 12 288 CpG island clones, 84 were shown to be hypermethylated in 70% of the patients with invasive bladder cancer as compared to their respective normal urothelium (Figure 1B). Figure 1C summarises known genes among hypermethylated gene promoters in bladder cancer, indicating the number of cases found differentially methylated with a Cy5/Cy3 ratio higher than 2 for these genes. A complete expanded version of the 84 clones is provided as Supplementary Table 1. Among these 84 hypermethylated clones with a Cy5/Cy3 ratio higher than 2.0 in the tumour specimen of at least 7 of the 10 patients, *SOX9* was selected for further validation analyses. This gene was chosen once we observed a significant statistical association of its decreasing transcript levels with increasing tumour stage (*P*<0.005) after analysing a previously reported transcript profiling series of our group (Sanchez-Carbayo *et al*, 2006).

### *SOX9* CpG island hypermethylation and its association with transcriptional gene silencing in bladder cancer cell lines

**Figure 2:**
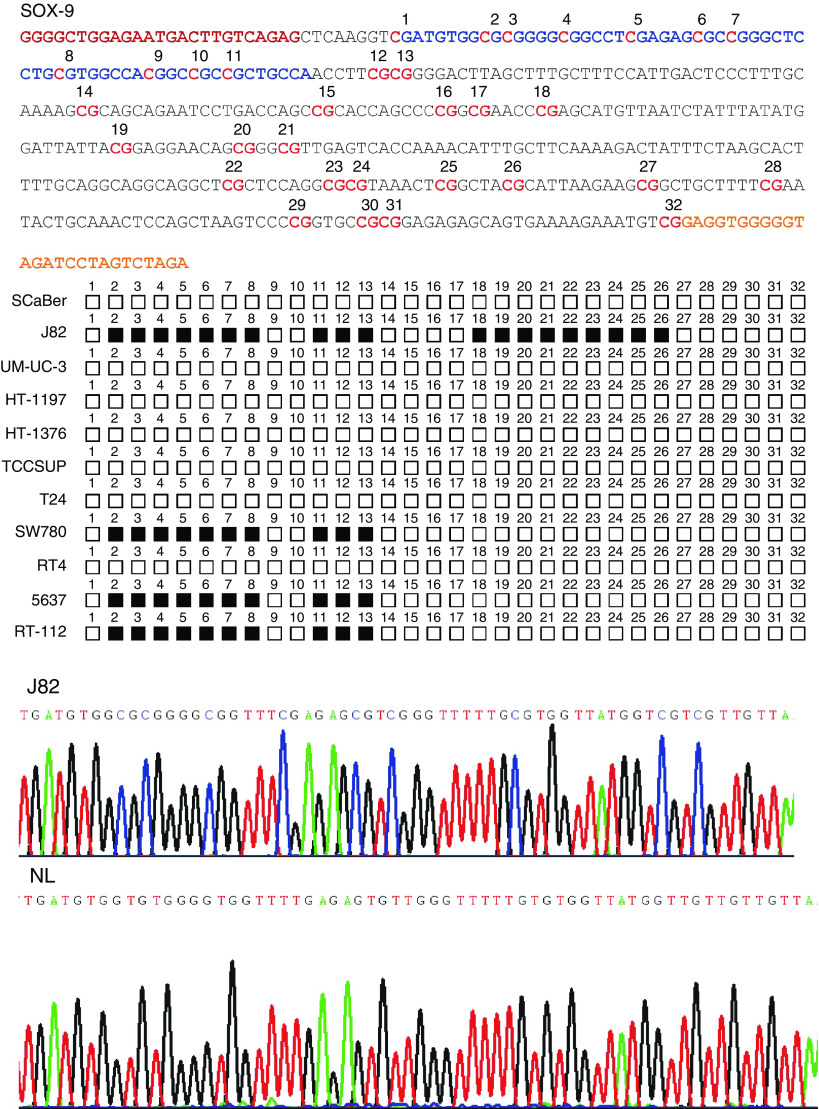
Analysis of CpG island methylation status of the promoter of *SOX9* by bisulphite genomic sequencing in bladder human cancer cell lines (*n*=11; including nonmuscle invasive, invasive, metastatic transitional and squamous cells). The upper part indicates the nucleotide sequences of the CpG island region analysed by bisulphite sequencing, sequencing primers highlighted in yellow and red and the area amplified in the chromatograms in blue. The mid-section shows a schematic depiction of the *SOX9* CpG islands around the transcription start sites. CpG dinucleotides are represented in squares. The presence of ‘Cs’ in the dinucleotide CpG reflects methylated cytosines (black squares), while the presence of ‘Ts’ in the dinucleotide CpG reflects unmethylated cytosines (white squares). Cell lines with black squares indicate the presence of methylation confirmed in at least two of the clones that were sequenced for each of the cell lines under analyses. The bottom part displays representative examples of the chromatograms obtained by bisulphite genomic sequencing of human cancer cell lines (a magnified boxed fragment is displayed). Normal lymphocytes (NL) were used as a negative sequencing control.

*SOX9* was tested to be a candidate gene for hypermethylation-associated inactivation in bladder cancer cells. Enriched 5′-CpG islands were found to be located around their transcription start sites, supporting their susceptibility to be epigenetically modified. To assess their methylation status, 11 human bladder cancer cell lines were initially screened using bisulphite genomic sequencing and MS-PCR targeted to the areas surrounding their transcription start sites. Bisulphite sequencing of these cell lines revealed CpG island methylation for *SOX9* (Figure 2). Among the normal tissues analysed, lymphocytes (NL) and urothelium were found unmethylated at the *SOX9* CpG island promoter (Figure 2).

**Figure 3:**
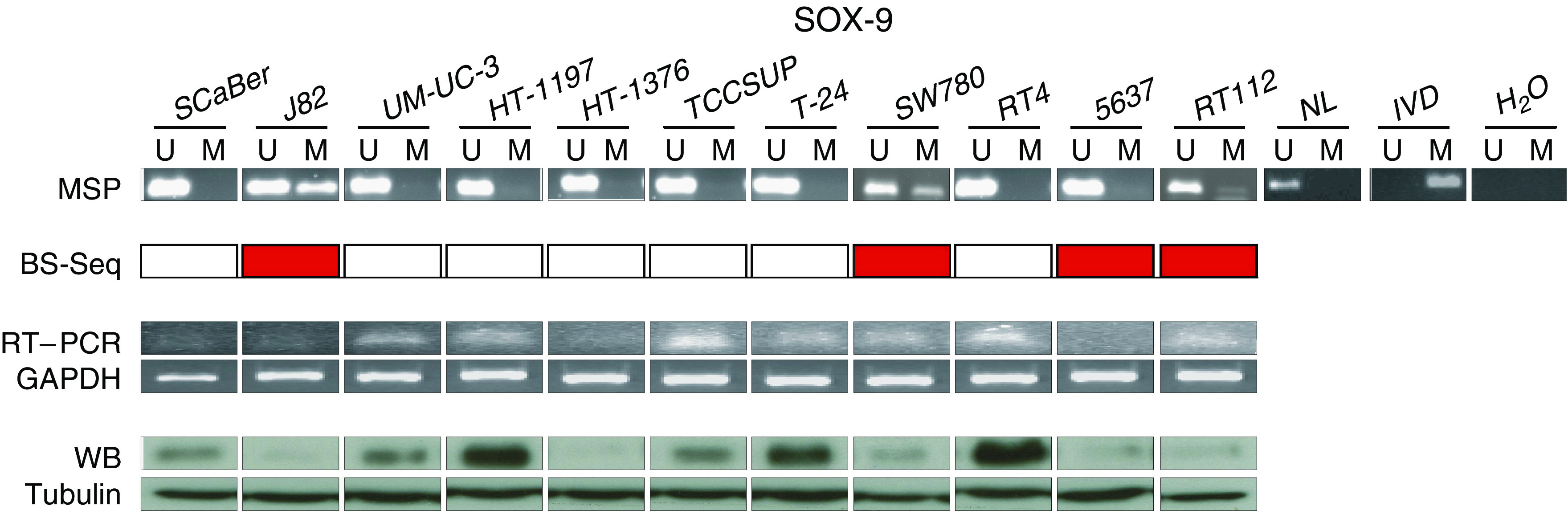
CpG island methylation is associated with gene silencing of *SOX9*. The upper part shows methylation-specific PCRs for *SOX9* in human bladder cancer cell lines. The presence of a PCR band under the lane M indicates a methylated gene, while the presence of a PCR band under the lane U indicates an unmethylated gene. Normal lymphocytes (NL) and *in vitro*-methylated DNA (IVD) were used as negative and positive controls for unmethylated and methylated PCRs, respectively. Sequencing information is included as well, highlighting methylated cell lines by genome sequencing in dark grey. Reverse transcription polymerase chain reaction analysis of SOX9 expression is displayed. Glyceraldehyde-3-phosphate dehydrogenase (GAPDH) transcript expression was used as a transcript loading control. Western blot analysis of protein expression is also shown. Tubulin expression was used as a protein loading control. The hypermethylated cell lines show relatively low transcript and protein expression of the coded protein as compared to unmethylated cell lines.

Having observed promoter hypermethylation in bladder cancer cell lines by bisulphite sequencing, specific MS-PCR was performed for *SOX9* (Figure 3). Methylation patterns observed by bisulphite sequencing highly correlated with the results obtained by MS-PCR, with differences attributable to the use of primers in these techniques encompassing slightly different areas around the transcription start sites for both strategies (Figure 2). Methylation analyses were then linked to transcript and protein expression estimates of the gene under study. The association between these epigenetic aberrations and putative transcriptional inactivation of this gene was initially assessed at the RNA and protein levels. Bladder cancer cell lines hypermethylated for *SOX9* showed low transcript and protein expression as revealed by RT–PCR and western blot analyses (Figure 3).

**Figure 4:**
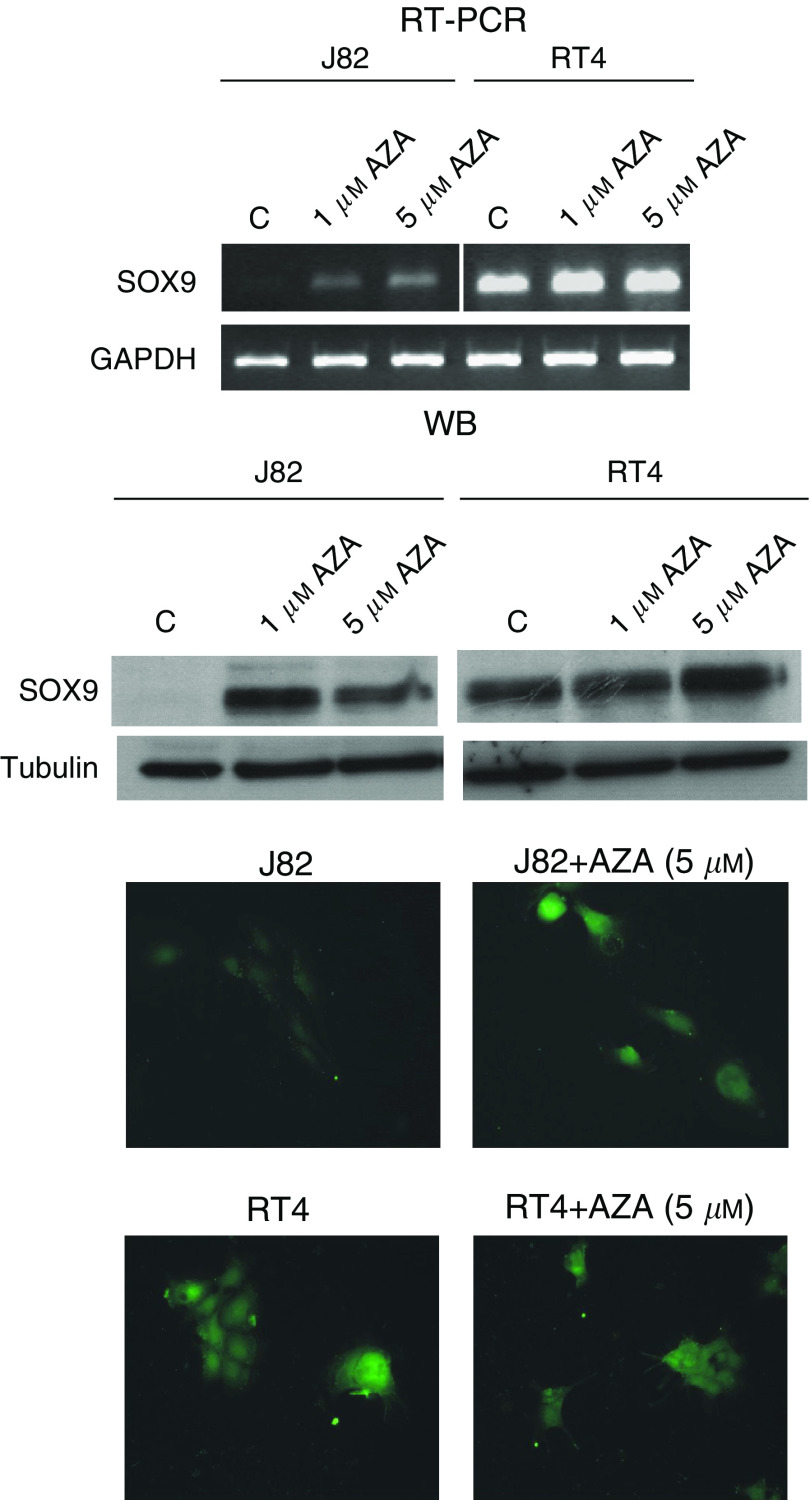
The treatment with the demethylating agent AZA reactivates gene expression of *SOX9*. The upper part displays the reverse transcription polymerase chain reaction analysis of *SOX9* expression. Glyceraldehyde-3-phosphate dehydrogenase (*GAPDH*) expression was used as a transcript loading control. The hypermethylated J82 cell line did not express *SOX9*, and restored *SOX9* transcript expression after AZA exposure. The mid-section shows western blot analysis of protein expression. Tubulin expression was used as a protein loading control. The hypermethylated cell line did not express the coded protein. The treatment with the demethylating agent reactivated *SOX9* protein expression. The unmethylated RT4 cell line did not show changes in transcript or protein expression. The bottom part displays immunofluorescence analysis of *SOX9* expression after AZA exposure. The methylated cell line did not show any staining for the protein, while the unmethylated ones showed its characteristic staining pattern.

Treatment of methylated and unmethylated bladder cancer cell lines with a DNA-demethylating agent served to further link *SOX9* hypermethylation and gene silencing. Exposure of methylated bladder cancer cell lines to the demethylating drug, AZA, restored expression of *SOX9* at the transcript level in the J82 cell line. RT4 was used as the control cell line to assess the specificity of AZA exposure, but not to modify gene expression of this candidate gene in unmethylated bladder cancer cells (Figure 4). Western blot and immunofluorescence analyses were performed to confirm that protein expression was also restored after AZA exposure. Overall, the results indicated a high correlation of methylation data with gene expression, observations especially supported by AZA reactivation analyses.

### *SOX9* is frequently hypermethylated in primary bladder tumours, and associated with clinicopathological variables

**Figure 5:**
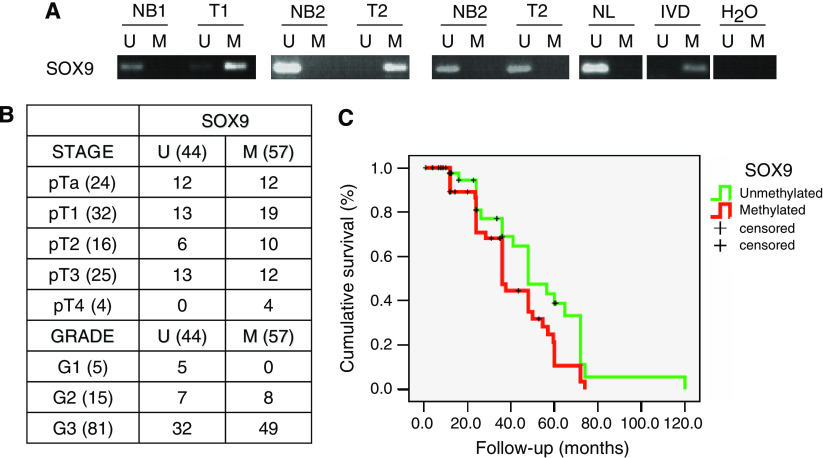
Association between *SOX9* hypermethylation with cancer progression and clinical outcome in bladder tumours. (**A**) Representative pairs of normal urothelium (NB) and primary bladder tumours (T) analysed by MS-PCR for *SOX9*. The presence of a PCR band under the lane M indicates a methylated gene, while the presence of a PCR band under the lane U indicates an unmethylated gene. Normal lymphocytes (NL) and *in vitro*-methylated DNA (IVD) are used as negative and positive controls for unmethylated and methylated PCRs, respectively. (**B**) Summary of the number of unmethylated (U) and methylated (M) cases for *SOX9* regarding their tumour stage and tumour grade. (**C**) Kaplan–Meier curve describing the association of *SOX9* hypermethylation with poor overall survival.

Once the functional consequences of *SOX9* CpG island hypermethylation were determined *in vitro*, it was tested whether hypermethylation of this gene was cancer-specific. Comparison of methylation of bladder tumours and their respective pairs of normal urothelium was analysed on an independent set of 10 cases for which paired normal urothelium DNA was available. Methylation was found in 70% of the bladder tumours and 10% of the normal urothelium tested. Illustrative examples of MS-PCR results among bladder tumours and paired normal urothelium are shown in Figure 5A. The relevance of their methylation in human clinical material was further analysed by MS-PCR in an independent large set of 101 primary bladder tumours. Hypermethylation patterns of this gene among these samples can be assessed in Figure 5B. Overall, *SOX9* CpG island hypermethylation was respectively found in 56.4% of the cases (57/101). The next analyses dealt with evaluating the link between the hypermethylation status of *SOX9* and clinicopathological variables of bladder cancer patients, as summarised in Figure 5B. Overall, tumours displaying high grade were more frequently methylated than those with low grade. A significant association of hypermethylation with tumour grade was found for *SOX9* (Mann–Whitney, *P*=0.032). Interestingly, *SOX9* hypermethylation was significantly associated with shorter overall survival (log-rank, *P*=0.025; Figure 5C). Therefore, *SOX9* hypermethylation was identified to be a likely predictor of poor outcome.

## DISCUSSION

The use of high-throughput profiling approaches is accelerating the discovery of genetic and epigenetic events associated with tumorigenesis and tumour progression. This study represents the first report describing the use of CpG island arrays as a means to comprehensively identify hypermethylation candidates in bladder cancer. By using a comprehensive approach, hypermethylation of novel genes was identified along bladder cancer progression due to the strategy of comparing bladder tumours and normal urothelium counterparts. Among the genes showing twofold hypermethylation in at least 7 of 10 cancers, *SOX9* gene was chosen for further analysis. To our knowledge, there was no data suggesting that *SOX9* could be epigenetically modified by methylation in human cancer. This gene was chosen once we observed significant statistical associations of its decreasing transcript levels with increasing tumour stage (*P*<0.005) after analysing a previously reported transcript profiling data of our group (Sanchez-Carbayo *et al*, 2006), suggesting their involvement in bladder cancer progression. This clinical observation excluded the need to estimate multiple testing and false discovery thresholds for *SOX9*, a common strategy undertaken for candidate selection in high-throughput studies.

The consequences of CpG island hypermethylation of candidate genes in bladder cancer progression need to be assessed from the standpoint of mechanistic, biological and translational implications. Mechanistically, it is important to evaluate the cellular consequences of the methylation of the promoter of *SOX9* in bladder cancer cells. Several techniques were utilised to link methylation analyses with expression estimates of the gene under study. Methylation status and expression results of *SOX9* correlated to high extent among a variety of bladder cell lines representing the spectrum of bladder cancer progression *in vitro*. AZA exposure experiments confirmed the impact of methylation in the expression of this gene by specifically restoring their transcript and protein expression in methylated bladder cancer cells. Overall, *in vitro* analyses demonstrated that the expression of *SOX9* is aberrantly silenced by CpG island promoter hypermethylation in bladder cancer, observations especially supported by AZA reactivation analyses.

To the best of our knowledge, *SOX9*, the gene identified in the present report, had not been previously related to bladder cancer. *SOX9* is a transcription factor that is expressed in chondrocytes and several tissues, including the central nervous and urogenital systems (Huang *et al*, 2000; Soderstrom *et al*, 2002). *SOX9* has been shown to be relevant at distinguishing mesenchymal chondrosarcoma from other small blue round cell tumours (Soderstrom *et al*, 2002; Wehrli *et al*, 2003) and at modulating retinoid-mediated growth in breast cancer cells (Afonja *et al*, 2002). Growth and tumorigenicity suppression associated with *SOX9* has been found in prostate, breast and colon cells (Afonja *et al*, 2002; Drivdahl *et al*, 2004; Jay *et al*, 2005). The epigenetic silencing of *SOX9* may aid understanding as to how it contributes to tumorigenesis and tumour progression in such types of neoplasias. Future studies are also warranted to dissect such mechanisms in the context of bladder cancer.

More importantly, the translational implications of the discovery of the methylation of this novel candidate gene identified by the CpG array have also been addressed in this work. CpG arrays identified higher methylation rates of the potential methylation candidate genes in tumour specimens as compared to normal urothelium counterparts. It was then necessary to test whether *SOX9* methylation was a cancer-specific epigenetic event. An independent set of 10 pairs of bladder tumours and normal urothelium, similar to the set used for the discovery of explorative analyses, was utilised to validate the presence of cancer-specific methylation of *SOX9* by an independent method, MS-PCR. SOX9 methylation was found with rates higher than 70% in bladder tumours and lower than 20% in their normal urothelium counterparts, an observation suggesting that *SOX9* could be methylated in a cancer-specific manner. Methylation in normal urothelium by MS-PCR could be attributed to the presence of mixed subpopulations to a certain extent detectable by a PCR-based technique, or that methylation could be a very early cancer epigenetic event, especially detectable in phenotypically normal urothelium counterparts due to the field effect. The initial discovery of this methylated candidate gene by comparing bladder tumours and their respective normal urothelium counterparts on the CpG arrays together with these observations on an independent set of cases were consistent with the concept of cancer-specific hypermethylation. This is a critical issue that requires to be tested for any hypermethylated candidates since the CpG arrays identified methylation higher in tumour specimens than in normal counterparts, without necessarily implying this epigenetic event to be cancer-specific.

It should be mentioned that several qualitative and quantitative methodologies are available for the purpose of determining methylation status of tumour specimens (Esteller, 2007). We selected standard gel-based MS-PCR, which could be considered appropriate enough for our descriptive analyses aiming at distinguishing between methylated and unmethylated cases. Considering the issue of tissue heterogeneity, only the complete absence of a PCR band was reported as a negative result. Quantitative RT–PCR methodologies are known to show their main advantages for comparative quantitative methylation analyses especially in body fluids. MS-PCR analyses in bladder cancer cell lines and independent sets of bladder tumours showed highly varying hypermethylation rates. CpG arrays and MS-PCR in the discovery and validation sets of 10 pairs of tumours and normal urothelium revealed the presence of *SOX9* methylation in at least 70% of the invasive cases analysed. Methylation rates were closer to 60% in the larger series of bladder tumours, which confirmed that *SOX9* hypermethylation is a frequent event in bladder cancer. Moreover, this independent set of 101 bladder tumours served to explore the clinical relevance of the identified methylation for *SOX9*.

The association of *SOX9* hypermethylation with bladder cancer progression can be justified as follows: first, the experimental design of the discovery of this methylated candidate gene by comparing invasive tumours *vs* their respective normal urothelium associates it with bladder cancer progression, providing cancer specificity to this candidate confirmed using independent sets of pairs of tumours and normal urothelium; second, this gene was selected based on its statistical association with tumour stage using transcript profiling analyses of bladder tumours (*P*<0.005); third, it was shown that methylation of *SOX9* is a frequent event among bladder tumours using an independent larger series of patients with bladder cancer. The methylation rates of 55.3% of the nonmuscle invasive cases showed that this epigenetic modification is an early event in bladder cancer. Moreover, the significant association of *SOX9* methylation with clinicopathological tumour grade and survival further supported their association with cancer progression. Interestingly, the presence of *SOX9* hypermethylation was associated with overall survival, confirming its potential role as a prognostic marker in the clinical management of patients affected with uroepithelial tumours. Such finding meets concordance with the knowledge of the involvement of this gene in human solid neoplasias. Inactivation by hypermethylation of *SOX9*, a gene reported to be a potential tumour suppressor, would be justified to be a poor outcome prognosticator (Afonja *et al*, 2002; Wehrli *et al*, 2003; Drivdahl *et al*, 2004). Thus, the gene identified in the present work represents a novel methylated tumour suppressor candidate to be investigated in bladder cancer. In addition to clinicopathological stratification of patients with bladder cancer, a relevant translational point relates to treatment, since it poses the potential use of demethylating agents such as 5-azacytidine in bladder cancer, to reactivate CpG island-hypermethylated genes, as has been approved by the Food and Drug Administration for a preleukemic disease, the myelodysplastic syndrome (Esteller, 2007). In this new scenario, *SOX9* represents a candidate target gene supporting the potential use of this type of drugs to achieve their demethylation and reactivation in bladder tumours. Further research would identify target-specific demethylating agents due to the complexity of epigenetic events along tumour progression.

In summary, our study discovers novel hypermethylated candidate genes in bladder cancer. It provides a mechanistic explanation for the observed loss of SOX9 in uroepithelial malignancies, demonstrating transcriptional silencing by promoter CpG island hypermethylation in bladder cancer cell lines. Although the ultimate consequences of its epigenetic inactivation in the molecular biology of the tumour cell remain unknown, its hypermethylation emerges as a strong indicator of tumour progression and clinical outcome for bladder cancer patients. These data should encourage further research into its downstream biological impact in tumorigenesis and cancer progression and eventually as a potential therapeutic target to be addressed following an epigenetic approach.

## Figures and Tables

**Table 1:** Primer sequences and PCR conditions for bisulphite sequencing (SEQ), methylation- (MSP) and unmethylation-specific PCR (USP) and RT–PCR for *SOX9*

	**Sense primer (5′ → 3′)**	**Antisense primer (5′ → 3′)**	**Product size (bp)**	**Annealing temperature (PCR cycles)**
SEQ	GGGGTTGGAGAATGATTTGTTAGAG	TCTAAACTAAAATCTACCCCCACCTC	434	62 (40)
MSP	GGTAGGTAGGTTCGTTTTAGGC	TCTCTCCGCGACACCGAAAA	152	58 (34)
USP	GTAGGTAGGTAGGTTTGTTTTAGGT	CTCTCTCCACAACACCAAAAACT	152	58 (34)
RT–PCR	AGTACCCGCACTTGCACAAC	CGTTCTTCACCGACTTCCTC	178	60 (25)
